# Analysis of a Dengue Virus Outbreak in Rosso, Senegal 2021

**DOI:** 10.3390/tropicalmed7120420

**Published:** 2022-12-07

**Authors:** Idrissa Dieng, Mamadou Aliou Barry, Cheikh Talla, Bocar Sow, Oumar Faye, Moussa Moise Diagne, Ousseynou Sene, Oumar Ndiaye, Boly Diop, Cheikh Tidiane Diagne, Gamou Fall, Amadou Alpha Sall, Cheikh Loucoubar, Ousmane Faye

**Affiliations:** 1 Arboviruses and Haemorrhagic Fever Viruses Unit, Virology Department, Institute Pasteur de Dakar, Dakar 220, Senegal; 2 Epidemiology, Clinical Research and Data Science Department, Institute Pasteur de Dakar, Dakar 220, Senegal; 3 Ministry of Health, Direction of Prevention, Dakar 220, Senegal; 4 DIATROPIX, Institute Pasteur de Dakar, Dakar 12900, Senegal

**Keywords:** DENV-1, Rosso, NS1 RDTs, outbreak response, serotype replacement, re-introduction

## Abstract

Senegal is hyperendemic for dengue. Since 2017, outbreaks have been noticed annually in many regions around the country, marked by the co-circulation of DENV1-3. On 8 October 2021, a Dengue virus outbreak in the Rosso health post (sentinel site of the syndromic surveillance network) located in the north of the country was notified to the WHO Collaborating Center for arboviruses and hemorrhagic fever viruses at Institut Pasteur de Dakar. A multidisciplinary team was then sent for epidemiological and virologic investigations. This study describes the results from investigations during an outbreak in Senegal using a rapid diagnostic test (RDT) for the combined detection of dengue virus non-structural protein 1 (NS1) and IgM/IgG. For confirmation, samples were also tested by real-time RT-PCR and IgM ELISA at the reference lab in Dakar. qRT-PCR positive samples were subjected to whole genome sequencing using nanopore technology. Virologic analysis scored 102 positives cases (RT-PCR, NS1 antigen detection and/or IgM) out of 173 enrolled patients; interestingly, virus serotyping showed that the outbreak was caused by the DENV-1, a serotype different from DENV-2 involved during the outbreak in Rosso three years earlier, indicating a serotype replacement. Nearly all field-tested NS1 positives samples were confirmed by qRT-PCR with a concordance of 92.3%. Whole genome sequencing and phylogenetic analysis of strains suggested a re-introduction in Rosso of a DENV-1 strain different to the one responsible for the outbreak in the Louga area five years before. Findings call for improved dengue virus surveillance in Senegal, with a wide deployment of DENV antigenic tests, which allow easy on-site diagnosis of suspected cases and early detection of outbreaks. This work highlights the need for continuous monitoring of circulating serotypes which is crucial for a better understanding of viral epidemiology around the country.

## 1. Introduction

Dengue fever is caused by one of four dengue virus (DENV) serotypes, namely DENV1-4 [1]. The virus belongs to the *Flaviviridae* family and flavivirus genus and is known to be present in many tropical and subtropical areas around the world [2]. Due to associated morbidity and mortality, the virus is one of the major public health threats in these areas [3]. Infection with the dengue virus causes clinical manifestations ranging from malaria-like symptoms (dengue fever) to life-threatening diseases (Severe dengue) [4]. According to the World Health Organization (WHO), 390 million people are infected by dengue each year [5]. Cases range between 50 to 100 million each year, with a fatality rate of 1–5% [6,7]. The virus epidemiology is relatively well-known in most American and Asian countries, but in contrast, little is known for the African continent, where the virus has circulated since the 19th century [8,9]. The lack of information about the dengue virus in Africa is probably linked to low awareness of the virus, the absence of effective surveillance, the presence of other pathogens causing similar clinical manifestations (malaria and bacterial infections) and the lack of diagnostic tools [9]. Dengue outbreaks associated with distinct serotypes have been reported from Zanzibar, Burkina Faso, Mauritania, Djibouti, Mozambique, Mauritania, Cote d’Ivoire, and Senegal [10,11]. Overall, all known dengue virus serotypes have been detected in Africa [12]. In Senegal, the first dengue case was reported in 1970 in the Bandia area located in the Thies region [13]. Since then, many outbreaks and sporadic cases [14,15,16] have been reported periodically. Until 1999, the landscape of dengue virus circulation was dominated by the circulation of sylvatic DENV-2 in southern Senegal [17]. In 2009, a major change was noticed with the first circulation of DENV-3 in West Africa, causing a large urban outbreak affecting Dakar, Louga and Thies [15]. In 2015, a study on arboviral etiologies of Non-Malarial febrile illnesses (NMFI) using a portable laboratory based on Recombinase Polymerase Amplification (RPA) detected three dengue cases serotype 1 in the suburb of Dakar [18]. Between 2017–2018, unprecedented multifocal and multiserotype circulations of dengue virus were noticed in Senegal, affecting mainly regions such as Louga (2017; Co-circulation DENV1-2), Thies (2018; Co-circulation DENV1-3), Fatick (2018; Co-circulation DENV1 and DENV-3), Touba (2018; circulation DENV-3), Rosso (2017; circulation DENV-2) [16]. Overall, past studies reveal growing evidence of dengue hyperendemicity in Senegal [19,20]. For virus detection, several PCR assays are available [21,22,23]. NS1 antigenic detection was shown to be useful in previous studies [24]; however, in Africa, RDTs test has not been commonly used for dengue diagnosis despite their performance and easy deployment at the point of need [24,25].

From week 39 in 2021, an increase in consultation activities for suspected arboviruses, with a peak in week 41, was observed through sentinel sites of the 4S network [26], including the site in the Rosso area. Blood samples collected within 5 days from 10 suspected arbovirus cases from the sentinel site of Rosso were sent to and analyzed at the WHOCC of the Pasteur Institute in Dakar, which confirmed DENV circulation. Here we describe results from multidisciplinary field outbreak investigation using the NS1 Antigenic test to detect dengue infection, and confirmatory qRT-PCR and serological assays at the reference lab as well as full genome characterization of circulating DENV genomes followed by phylogenetic analysis of detected strains.

## 2. Materials and Methods

### 2.1. Study Design and Setting

#### 2.1.1. Syndromic Sentinel Surveillance Network in Senegal (4S Network)

Senegal has a longstanding surveillance system, initially focused on influenza virological surveillance in 1996, which then adopted a syndromic approach in 2011 through the establishment of the Senegalese Syndromic Sentinel Surveillance Network (4S network), a partnership between the Ministry of Health (MoH), the WHO country office and the Institut Pasteur of Dakar (IPD) [26]. In addition to influenza-like illness (ILI), other public health priority syndromes (malaria, dengue-like syndromes and diarrheal syndromes) have been added through an integrated approach. An early warning system (EWS) of diseases under surveillance was set up in 2015 that allows the MoH to detect and alert quickly to any abnormal health event. The 4S network provides ‘real-time data on dengue-like syndromes epidemiology across the country.

#### 2.1.2. Study Sites

Initially, the investigation was carried out in the coverage area of the sentinel site of Rosso 1 (located about 388 km from Dakar). Then, the investigation extended to other health structures in the Rosso area (Rosso 2 and Mbagam health posts are located about 5 & 15 km, respectively, from the sentinel site). To assess the scale of the epidemic at the health district level, the district hospital (the Richard Toll health centre with its ancillary structures) was also investigated.

### 2.2. Outbreak Investigation and Patient Enrolment

An outbreak investigation was conducted from 13–20 October 2021. The investigation team visited the different housing areas of confirmed cases that were identified through active door-to-door case searches.

A working case definition was developed, which was:

- Suspected case: sudden onset of fever (>38 °C) in a resident of the survey area, with two or more of the following signs/symptoms: headache, retro-orbital pain, arthralgia, myalgia, nausea/vomiting, rash, hemorrhagic manifestations, leukopenia.
- Probable confirmed case: Any suspected case with a positive Dengue RDT (IgM/IgG or detection of viral antigen NS1).
- Confirmed case: Suspect or probable case confirmed by the reference laboratory (positive IgM serology, increase in IgG titers, virus detection by RT-PCR or isolation on cell culture).

A structured questionnaire was used to collect socio-demographic data, date of onset of illness, signs/symptoms, risk factors information (mosquito exposure, use of bednets) and travel outside the investigation area 15 days prior to illness.

A blood sample (3 to 5 mL) was collected by venipuncture into a Vacutainer tube for each clinically dengue suspected case. The collected samples were transported in cooled boxes to the district health Laboratory of Richard-Toll.

### 2.3. NS1/IgM-IgG Rapid Detection Tests Detection on the Field

The SD biosensor Standard Q^TM^ Dengue Duo test (SD Biosensor, Yeongtong-gu, Korea) allows simultaneous detection of DENV NS1, IgM and IgG using a total of 110 µL of the sample (Whole blood, serum or plasma) [27,28]. To increase the sensitivity, collected sera samples were centrifuged at 4000 rpm for 2 min; for NS1 antigen detection, 100 µL of serum was added into the sample well. For IgM/IgG detection, 10 µL was added to the S area (S for sample) followed by 3 drops of diluent. Results of both tests were read by the naked eye after 15 min of incubation at room temperature according to manufacturer indications.

### 2.4. Molecular Diagnostics for DENV Detection and Serotyping

Sera from collected blood samples during the field investigation were aliquoted and temporarily conserved at −20 °C and shipped to the WHOCC for additional and confirmatory tests. For molecular detection of DENV among suspected cases, 140 µL of sera were used for RNA extraction using QiaAmp Viral RNA Extraction Kit (Qiagen, Heiden, Germany) according to the manufacturer’s instructions. DENV 3′UTR region was amplified by qRT-PCR using a set of primers previously described by [29] on a Quantstudio cycler (Thermo Fisher Scientific, Waltham, MA, USA) using AgPath-ID One-step RT-PCR kit (Thermo Fisher Scientific, Waltham, MA, USA).

Samples with Ct < 35 were considered positive. To determine the DENV serotype-positive samples by qRT-PCR, real-time molecular serotyping was performed on the RNA extracts using the commercial Tib-Molbiol Modular Dx Dengue Typing Kit (Cat-No. 40-0700-24) on the Lightcycler 480 (Roche, Penzberg, Germany) according to a previously published protocol [30]. This system allows the simultaneous detection of four dengue virus serotypes in one single reaction from 5 μL of input RNA.

### 2.5. Serological Assays for Specific DENV Antibodies Detection

In addition to molecular detection of DENV, collected sera were subjected to enzyme-linked immunosorbent assay (ELISA) for the detection of specific DENV IgM antibodies [31]. Due to recognized cross-reactivity among viruses belonging to the *flavivirus* genus, IgM-positive samples were confirmed by plaque reduction neutralization tests (PRNTs) for the dengue virus [31].

### 2.6. Sequencing and Phylogenetic Analysis

Whole genome sequencing of DENV was performed directly on RNA of positive cases using amplicon-based tilling PCR followed by sequencing using Nanopore technology. Briefly, RNA was used as a template for first-stranded cDNA synthesis using the Lunascript RT supermix kit (New England Biolab, County Road, Ipswich, MA, USA). An amplicon-based approach was then performed by using multiplex PCR primer schemes designed to amplify on two pools the entire coding region of DENV-1 [32]. RT-PCR reactions were undertaken using Q5 high fidelity 2X master mix (New England Biolabs, County Road, Ipswich, MA, USA), and PCR products were checked for expected amplicon size (around 900 bp) on a 1.5% agarose gel. Amplicons were purified using a 1:1 ratio of Ampure beads (Beckman Coulter). For sequencing, purified amplicons were tagged using the Rapid Barcoding Kit 96 (SQK-RBK110.96) according to the manufacturer’s recommendations. Water was used as a blank control sample. The obtained libraries were quantified, normalized, pooled and loaded on R9.4.1 flow cells (FLO-MIN106D) on the Oxford Nanopore MinION platform. The sequencing reads were base-called using guppy and merged to a single Fastq file, and consensus sequences were generated with genome detective (https://www.genomedetective.com/ accessed on 9 March 2022). To define the genotype of the newly assembled DENV-1, we used the genome detective dengue typing tool (https://www.genomedetective.com/app/typingtool/dengue/ accessed on 4 September 2022).

Nearly complete genomes obtained during this work (n = 6) were combined with a representative subset of DENV sequences available in Genbank (n = 64) sampled from Senegal, Africa and globally. The obtained final dataset (n = 70) was aligned using MAFFT [33]. A Maximum Likelihood (ML) tree was then drawn using IQ-TREE [34] by adding an automatic model selection argument using a model finder (MF) implemented on the software using the Bayesian Information criterion. Bootstrap values were set up to 1000 replicates for robustness. The obtained tree was visualized and annotated using R software and ITOL [35].

### 2.7. Statistical Analysis

Demographic, clinical characteristics and Diagnostic tools result from the comparison of the study population between suspected negatives and confirmed dengue cases were analyzed using χ2-test or Fisher’s exact test. Mean age comparison between suspected negatives and confirmed dengue cases was done using Kruskal–Wallis test. A *p*-value < 0.05 was considered statistically significant. The statistical analysis was performed with Stata version15 software (StataCorp, LLC, College Station, TX, USA)

### 2.8. Ethical Consideration

The Senegalese National Ethical Committee of the Ministry of Health approved the sentinel surveillance protocol approved by the CNERS through the Arbosen project (N°000243/MSAS/DPRS/CNERS). Oral consent to participate was obtained from all patients or parents of minors included in this survey. Throughout the study, the database was shared with the National Epidemiology Department at the Senegalese Ministry of Health for appropriate public health actions.

## 3. Results

### 3.1. Demographic Characteristics and Epidemiology

To study a Dengue virus outbreak in Rosso Senegal, a total of 173 serum samples were collected from different localities across Rosso Senegal and Richard Toll health district from suspected dengue fever cases between 13 October to 19 2021. Overall the DENV infection was 58.0% (102/173). The median age of all patients was 21 years (IQR: 11–37 years). Most confirmed cases were under 15 years (37.0%), followed by the 15–30-year-old (29.5%). The comparison between subjects with negative and positive dengue fever infection did not show any difference regarding the age groups (*p* = 0.97). Among the suspected dengue fever cases identified and interviewed, 89 (51.4%) were females, and 84 (48.6%) were males. M:F sex ratio for the positive cases was 1.2 (56 males vs. 46 females). There was a significant DENV positivity rate difference according to gender (*p* < 0.05). The most common symptoms reported were fever recorded in all suspected cases, followed by headaches (74.0%), arthralgia (62.0%) and myalgia (59.0). For the confirmed cases, 71.6 % (n = 73/102) were from the sentinel syndromic surveillance site at Rosso 1 and 20.6 % (n = 20/102) from Mbagam PHC with significant difference between suspected and positive cases (*p* < 0.001) (Table 1).

### 3.2. Epidemiological Curve

**Figure 1:**
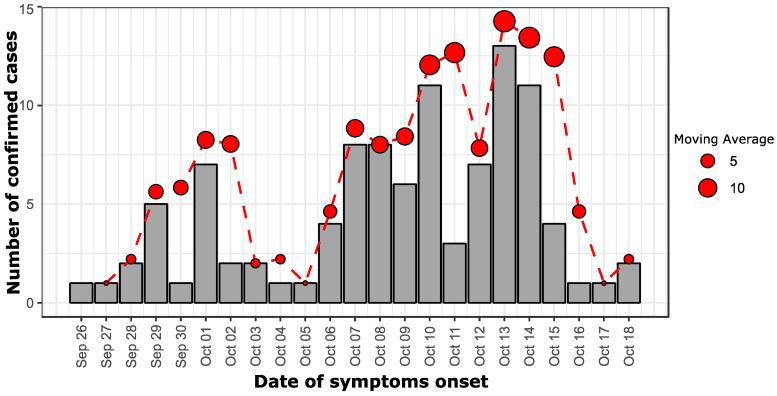
An epidemiological curve showing the number of confirmed dengue cases during this outbreak investigation according to the date of symptoms onset; a dashed red curve shows the moving average of confirmed dengue cases per 2 days. The size of the dot is proportional to the value of the moving average.

The Epidemic curve showed that the first case had the date of onset of illness on 26 September 2021 and most of the cases developed signs/symptoms on 1 October (n = 7; alert), 7–10 October (n = 33; 2nd wave) and 12–15 October 2021 (n = 35; 3rd wave) (Figure 1)

### 3.3. Circulating Dengue Virus Serotypes

To determine the serotype of positive cases, we performed serotyping using the Modular Dx Dengue Typing qRT-PCR. Among 64 DENV-positive samples, 50 were successfully serotyped as DENV-1; 14 failed to be serotyped. DENV-2,-3 or-4 were not detected (Table 2).

### 3.4. Comparison of NS1 vs. qRT-PCR Performance

**Figure 2:**
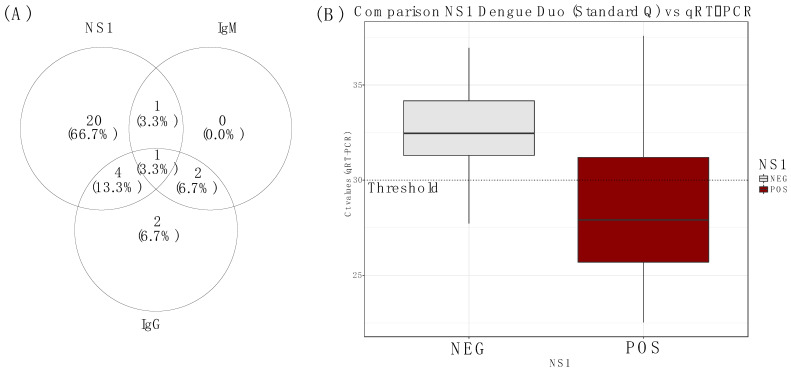
(**A**) Venn diagram showing repartition of Standard Q NS1/IgM/IgG positive test results from the field; the number of samples positives DENV according to the targeted analyte (NS1,IgM and IgG), (**B**) Box plot showing a comparison of the median Ct values of Standard Q DENV NS1/IgM/IgG RDT on NS1-positive and NS1-negative samples.

To investigate the performance of an RDT for the detection of dengue NS1/IgM/IgG, 70 samples collected in the field investigation were screened for dengue NS1/IgM/IgG by rapid diagnostic test on site. All collected sera samples were then tested at the laboratory using qRT-PCR. Among them, 26 samples were positive for DENV NS1 antigen, 4 samples for IgM (n = 2/4 were also positive for NS1 antigen), and 10 samples were positive for IgG (Figure 2A). Interestingly, 24/26 NS1 positives were also positives by real-time RT-PCR at the reference lab. A comparison of both tests (NS1 vs. qRT-PCR) shows that the mean Ct values were significantly higher on the NS1 positive sample when compared to the NS1 negative (Figure 2B).

### 3.5. Phylogenetic Inference

**Figure 3:**
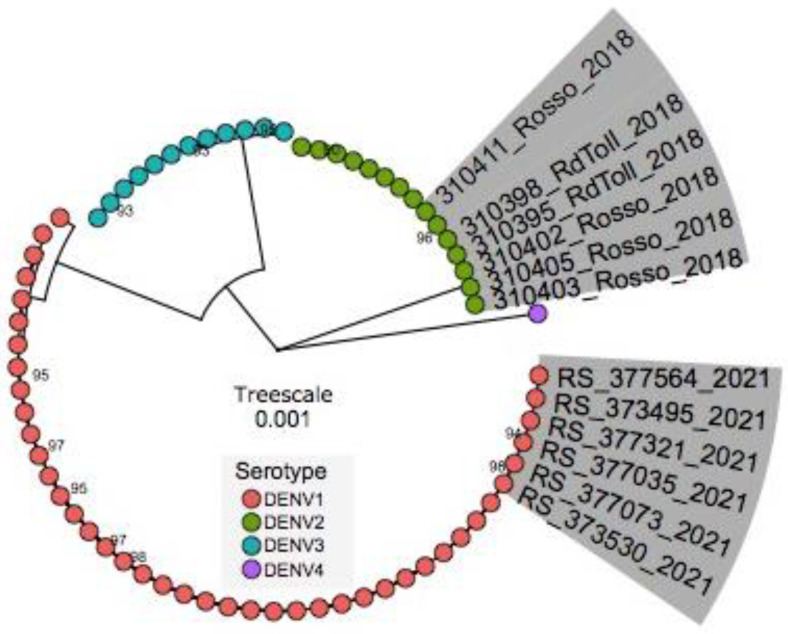
Maximum Likelihood tree showing the relationship and genetic diversity of Senegalese DENV-1 strains detected during this study. Sequences in this study and publicly available sequences belonging to described DENV serotypes were used. Tips are colored according to the serotype. Sequences of Rosso 2021 (this study) and those from a previous outbreak in 2018 are highlighted in grey. The tree was built using complete genomes. Only the names of Rosso sequences (RS) (2018 and 2021) are represented; the designation of all used sequences is depicted in Figure S1 (supplementary files).

The Maximum Likelihood (ML) tree revealed that the sequences obtained during the investigation belong to DENV1/Genotype V but fall in a cluster different from strains isolated during previous DENV1/Genotype V outbreaks in Senegal. It confirmed that during this outbreak, DENV1 was the serotype responsible for the epidemic (Highlighted in grey with Rosso2021 caption) and not DENV2/Cosmopolitan genotype, which was detected in the Rosso outbreak in 2018 (Highlighted in grey with Rosso2018) (Figure 3). Interestingly it showed that the circulating strain is more closely related to strains detected in Burkina Faso in 2017 (MW243050) and Cote d’Ivoire in 2019 (MW243052) than to DENV1 detected during an outbreak in Louga Area in 2017 (Figure S1; Raw Tree).

## 4. Discussion

As shown in previous studies, dengue is hyperendemic in Senegal [16,36]. Many epidemics caused by different serotypes were reported in many regions, including Louga in 2017 (DENV-1), Fatick in 2018, Touba in 2018 (DENV-3), Thies in 2018 (DENV1-3), Rosso in 2018 (DENV-2) by our group [16]. Since 2017 molecular monitoring of circulating serotypes was implemented in Senegal at the WHOCC for arboviruses and haemorrhagic fever viruses, establishing a basic molecular database for a better understanding of molecular epidemiology, the introduction of new as well as spread and dispersal patterns of known circulating serotypes.

Here we report a DENV-1 outbreak at Rosso (Northern Senegal) in 2021 and the findings from the field investigation involving a multidisciplinary team; this study was also an opportunity to test the reliability and the usefulness of a DENV NS1 antigenic test during outbreak response for the first time in Senegal. The outbreak was monitored over 8 weeks, and 173 blood samples were collected from suspected cases. Performed virologic tests on collected human samples during outbreak investigation revealed a DENV prevalence of 58.95 % (qRT-PCR and IgM).

Molecular serotyping of qRT-PCR positives (n = 50) was confirmed to be DENV-1 suggesting this serotype replaced DENV-2, previously responsible for the 2018 outbreak in Rosso (Unpublished data). For dengue infection, it is well known that infection with one serotype induces long-lasting immunity against this particular serotype but does not protect against heterotypic infection [37,38]. Compared to the 2018 DENV outbreak, where the positivity rate was 19.77%, a higher number of infections was noticed during this study. This high prevalence is most likely due to the introduction of DENV1 to the population serological naive for this Dengue serotype which can be associated with a higher number of symptomatic and severe cases [39,40]. Circulation of a new serotype or genotype in a region may cause bottlenecks and displacement of previously dominant serotypes, but both serotypes can continue to co-circulate [41]. The sequential circulation of different Dengue virus serotypes in a given area can be associated with a higher incidence of severe dengue manifestations due to the antibody-dependant enhancement phenomenon [42,43]. Surprisingly despite the sequential circulation of different DENV serotypes in Rosso between 2018 and 2021, all detected cases are classified as DwoWS. These findings are inconsistent with a previous study performed in India [44] and Peru [45], where the introduction of a new lineage of DENV-2 was associated with increased severe dengue manifestations. Overall it is well known that the introduction of a new group of viruses in a population with serological naivety [45,46] or previously exposed to a different serotype [47] has the potential to cause unprecedented outbreaks with a higher number of severe cases. Despite the occurrence of a new serotype, the implication of other factors such as urbanization, better transportation, and increased adaptation of *Aedes spp* on virus spread cannot be ruled out [48]. The Rosso region shares a border with the southern part of Mauritania, a region where the presence of *Aedes aegypti* has been documented since the 1960s [49].

During the epidemiological investigation, the local population reported a higher density of mosquitoes in recent years in Rosso, which coincided with the beginning of urbanization which may have facilitated the creation of artificial breeding sites. An increase in vector density is known to increase arboviral transmission [50].

Dengue NS1 circulation in the blood exceeds viremia circulation [51]. Indeed previous studies [52,53] revealed that soluble NS1 (sNS1) could persist in the blood of infected patients for 9 days. During the field investigation, 70 sera samples were tested for DENV NS1/IgM/IgG yielding 26 NS1 antigens, 92.3% of which were concordant with qRT-PCR results. Thus the detection of NS1 constitutes a good alternative to qRT-PCR during an outbreak investigation of acute cases. Interestingly, NS1 positive cases showed significantly lower median Ct values (*p* = 0.0068; *t*-test), indicating higher DENV viremia than NS1 negative samples. This finding confirms results from previous work suggesting that NS1 positivity is linked to viral load. No associations were found between dengue infection and age group (*p* = 0.97; *χ2-*test); however, dengue positivity was significantly associated with sex (*p* = 0.04; *χ2-*test) with males (56/102; 54.9%) more prone to dengue infections than females (46/102; 45.1%). This trend is different to the pattern observed during the dengue fever outbreak in Louga in 2017, where an equal rate of dengue positivity across both sexes was observed [36] but consistent with findings observed elsewhere [54,55,56]. The high occurrence of dengue infection in males compared to females is probably linked to the fact that males are prone to more outdoor activities, which increases their chance of being bitten by infected mosquitoes [57].

Phylogenetic analysis grouped the epidemic DENV1 genogroup V strain into a different cluster than the strain detected in the Louga area in 2017. This points to a probable re-introduction event of this genotype to Senegal instead of sustained endemic circulation in Senegal over the years. In contrast, a previous full E gene sequence analysis revealed a unique introduction followed by dispersion of DENV1/Genotype V in Senegal between 2015 to 2019 [30]. These findings call for in-depth phylogeographic analysis to elucidate the origin and spread of this particular genotype after 2019.

This study collected evidence on the co-circulation of many DENV serotypes in Senegal and the hyperendemic behavior of the virus around the country. It also highlighted the potential repeated virus introduction, which is a hallmark of regional viral transmission across bordering countries. Additionally, it showed the usefulness of NS1 RDTs during outbreak response and allowed to advocate a large-scale implementation of this tool for early detection of DENV cases and a large-scale deployment at the point of need.

Overall, during this DENV outbreak investigation in the Rosso area, we evaluate for the first time in Senegal the deployment of DENV RDTs for epidemic response purposes constitutes an important and relevant public health tool with a positive impact on early cases management and diagnosis at the point of need. Despite the reliability, some limitations associated with the study need to be raised. First, during the study, we only tested on the field a subsample among suspected cases for NS1 antigen using RDTs (n = 70); not all suspected cases were processed with as a consequence, a delay in the investigation of additional cases detected at the central lab using qRT-PCR. Secondly, among suspected cases, 41.04% of cases were not linked to DENV infection. Other pathogens associated with the same clinical manifestations as malaria, other arboviruses or bacterial infections can co-circulate this call for systematic differential diagnosis or metagenomic approach on these samples with a consequence avoiding cryptic outbreaks that are caused by viruses and/or bacteria with similar clinical signs. Thirdly since this study raised the occurrence of serotype replacement, the primary or secondary infection status of collected samples was not analyzed during this work; this constitutes a major limitation, and it will be interesting to asses this with additional study.

## Figures and Tables

**Table 1:** Summary of epidemiological & clinical characteristics of suspected and confirmed dengue fever cases investigation, Rosso Senegal, 2021.

	Total (n = 173)	Positives (n = 102)	Negatives (n = 71)	*p*_Value
	n (%)	n (%)	n (%)	
Median (IQR ^1^)	21 ± (11–37)	21 ± (9–36)	23 ± (11–39)	0.49 ^a^
Age group (years) <15 15–30 30–45 >=45	64 (37.0) 51 (29.5) 25 (14.5) 33 (19.1)	39 (38.2) 30 (29.4) 14 (14.0) 19 (18.6)	25 (35.2) 21(30.0) 11 (15.5) 14 (20.0)	0.97 ^b^
Sex Female Male	89 (51.4) 84 (48.6)	46 (45.1) 56 (54.9)	43 (60.6) 28 (39.4)	0.04 ^b^
Headache Yes No	128 (74.0) 45 (26.0)	82 (80.4) 20 (19.6)	46 (65.0) 25 (35.2)	0.02 ^b^
Myalgia Yes No	101 (59.0) 70 (41.0)	64 (63.4) 37 (36.6)	37 (53.0) 33 (69.0)	0.17 ^b^
Arthralgia Yes No	107 (62.0) 66 (38.0)	69 (67.6) 33 (32.4)	38 (53.5) 33 (46.5)	0.05 ^b^
Asthenia Yes No	64 (37.0) 109 (63.0)	37 (36.3) 65 (63.7)	27 (38.0) 44 (62.0)	0.81 ^b^
Abdominal pain Yes No	8 (4.6) 165 (95.4)	4 (4.0) 98(96.0)	4 (5.6) 67 (94.4)	0.60 ^b^
Retro-orbital pain Yes No	3 (2.0) 170 (98.0)	2 (2.0) 100 (98.0)	1 (1.4) 70 (98.6)	0.63 ^b^
Investigated health structures Rosso 1 (4S site) Mbagam PHC ^#^ Richard Toll DHS” Rosso 2 PHC ^#^	99 (57.2) 55 (32.0) 10 (6.0) 9 (5.2)	73 (71.6) 21 (20.6) 2 (2.0) 6 (6.0)	26 (36.6) 34 (48.0) 8 (11.3) 3 (4.2)	<0.001 ^b^

PHC ^#^: Mbagam Primary Health Care, DHS”: Richard Toll District Health Structures, IQR ^1^: Inter Quartile Range, ^a^: stands for the Kruskal-Wallis test, ^b^ stands for *χ2-*test or Fisher’s exact test.

**Table 2:** Repartition of detected DENV serotypes among qRT-PCR+ samples.

Molecular Diagnostic	n (%)	95 % CI
qRT-PCR all DENV	64/176 (36, 36%)	29.25–43.47
DENV-1	50/64 (78, 12%)	67.99–88.25
DENV-2	00/64	NA
DENV-3	00/64	NA
DENV-4	00/64	NA
Co-infections	00/64	NA

## Data Availability

The data that support the findings of this study are available from the corresponding author upon reasonable request.

## Supplementary Materials

The following supporting information can be downloaded at: https://www.mdpi.com/article/10.3390/tropicalmed7120420/s1, Figure S1: Phylogenetic Tree of detected DENV-1 and DENV-2 in Senegal between 2017 to 2021.
