# Supplementary figures and images for: Analysis of a Dengue Virus Outbreak in Rosso, Senegal 2021
Source: Trop Med Infect Dis. 2022 Dec 7;7(12):420. doi: 10.3390/tropicalmed7120420 (PMC9781526; doi:10.3390/tropicalmed7120420)

Tree scale: 0.1

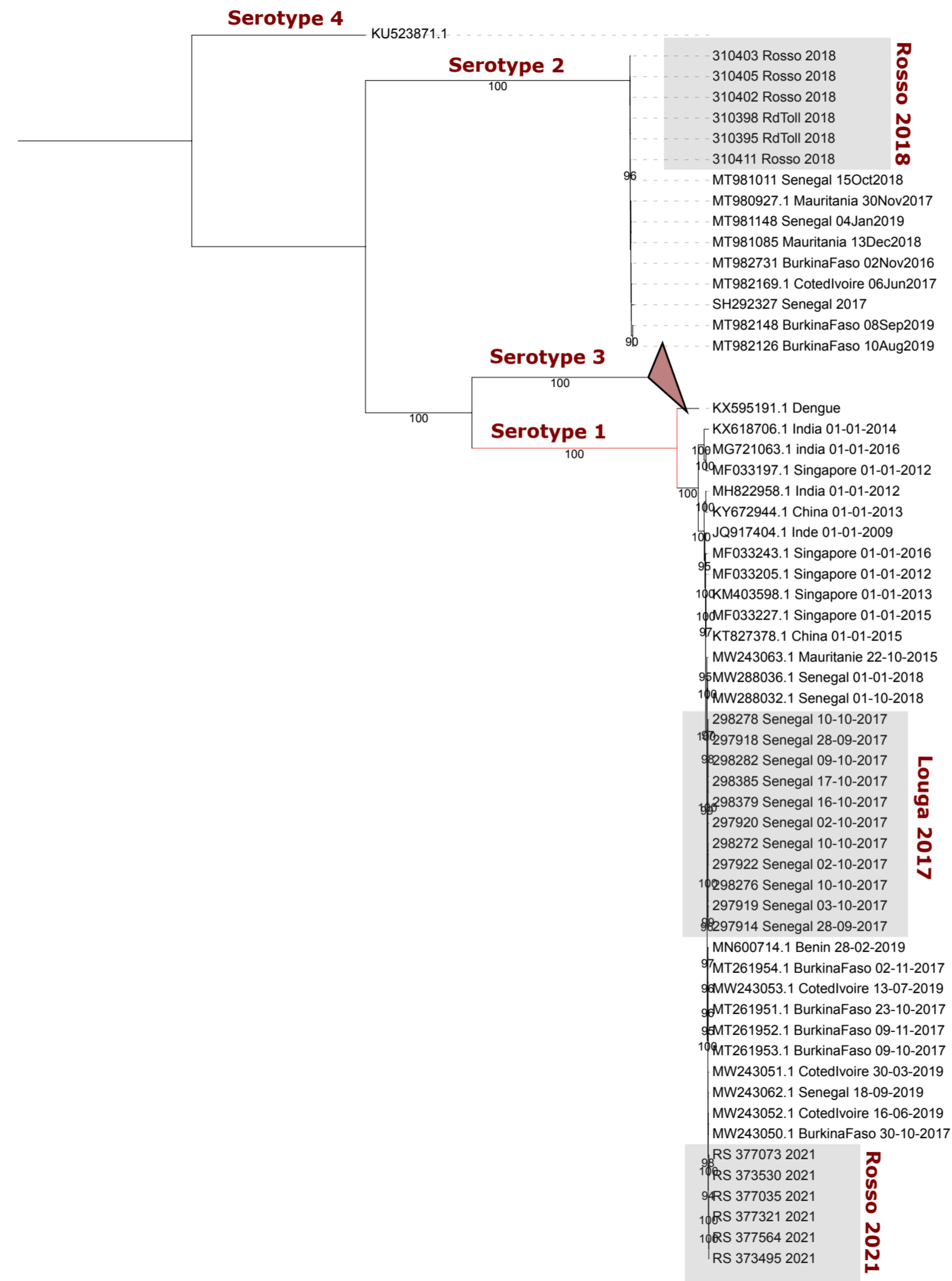

**Figure S1:** Phylogenetic Tree of detected DENV-1 and DENV-2 in Senegal between 2017 to 2021.

Supplement: Supplementary file 1 [file tropicalmed-07-00420-s001.zip › tropicalmed-1991293-supplementary.pdf]
